# The Dose-Response Efficacy of Physical Training on Frailty Status and Physical Performance in Community-Dwelling Elderly: A Systematic Review and Meta-Analysis of Randomized Controlled Trials

**DOI:** 10.3390/healthcare10030586

**Published:** 2022-03-21

**Authors:** Pei-Shan Li, Chia-Jung Hsieh, Eva Berthy Tallutondok, Hsuan-Ju Peng

**Affiliations:** 1 School of Nursing, College of Nursing, National Taipei University of Nursing and Health Sciences, Taipei 112303, Taiwan; phli2@vghtpe.gov.tw (P.-S.L.); eva.tallutondok@uph.edu (E.B.T.); jiazi1230@hotmail.com (H.-J.P.); 2 Department of Nursing, Taipei Veterans General Hospital, Taipei 112303, Taiwan; 3 Faculty of Nursing, Universitas Pelita Harapan, Banten 15811, Indonesia; 4 Department of Nursing, Taoyuan Armed Forces General Hospital, Taoyuan 325208, Taiwan

**Keywords:** community elderly, frailty, physical training, physical performance

## Abstract

Purpose: This systematic review and meta-analysis was conducted to explore the effect of physical training on frailty status and physical performance in the community dwelling elderly. Methods: We set keywords and used the Boolean operator to search the CEPS, CINAHL, Cochrane Library, PubMed, MEDLINE, and EMBASE databases from inception to 10 August 2021. The search was limited to randomized controlled trials (RCTs) conducted within a five-year period. The Cochrane Collaboration bias assessment tool was used to assess article quality, and RevMan 5.4.1. software (Cochrane Training site based in London, UK) was used to conduct the meta-analysis. Results: Physical training was found to improve frailty status, physical performance, lower limb strength and balance. The best dose-response for physical training was 60 min per time, 2–3 times per week, for 3 months. Conclusion: Designing an appropriate physical training program can decrease the frailty score and increase physical performance in frail elderly in the community.

## 1. Introduction

The aging of the world’s population is an irreversible situation because of the rapidly increasing number of the elderly in the 21st century [1]. The physical activity of the elderly population is often limited to standing up from a seated position or contracting muscles to adapt to changes in the internal and external environment. However, physical activity is an important factor related to healthy behavior and health promotion [2]. When the physiological or organ function of the elderly gradually undergoes senescence, physical and mental states advance to the frail state as age progresses [3]. Older frail adults also experience muscular weakness, fatigability, a slow walking speed, sedentary lifestyle, and unintentional weight loss. [4]. It represents an increased risk of adverse aging-related physical function and health problems [5]. Therefore, physical performance is an indicator of the health status of the elderly.

More RCTs have taken place in recent years, with more researchers showing an interest in physical training within community-dwelling elder care [6]. Research has shown that physical activity can improve frailty status and physical performance in the community-dwelling elderly [7]. The value of physical activity for older adults has been widely acknowledged [8], and existing systematic reviews that a focus on the physiological conditions of the community-dwelling frail elderly shows that physical training can improve physical function [6,9,10,11]. Worldwide multi-dimensional research has focused on frailty prevention in communities or groups and is usually based on physical training, which includes muscle endurance, balance and cardiovascular activity [12,13,14,15]. For dose-responses in physical training, optimal-dose, frequency, and duration parameters are critical for developing an appropriate program [16]. However, the dose-response efficacy of physical training in the elderly on physical performance and frailty status is still unclear and has not yet been evaluated. This results in incomplete information on the dose, frequency, and duration of physical training. The community-dwelling frail elderly who have excessive or insufficient physical training are unable to improve their physical function or may develop physical injuries. However, previous studies that focused on optimal dose-response in physical training on physical performance and frailty were missed [7]. Therefore, the best physical training design should include dose, frequency and duration criteria to reduce or prevent the risk of injuries so that the community-dwelling frail elderly can receive the most benefit.

The relevant literature does not provide a consistent recommendation on the dose-response of physical training in the community-dwelling frail elderly. Knowledge of the optimal dose-response of physical training can inform program development, improve compliance, and increase the confidence of planners and participants. Exploring the dose-response to clarify the physical training load and examining the results of physical performance will promote the development of the best care model and develop evidence that supports health promotion. This systematic review integrates randomized controlled trials (RCTs) of physical training in the community-dwelling frail elderly and gives an overview of the different dose-response efficacies of physical training on physical performance and frailty status. The results of this research should lead to improvements in the inferences of future studies, advances in research programs, and the construction of more complete care programs for the frail elderly population.

## 2. Methods

### 2.1. Literature Search

This research was conducted from 10 August 2021 to 22 October 2021. Searches were performed in one Chinese electronic database, the CEPS, and five English electronic databases: CINAHL, Cochrane Library, PubMed, MEDLINE and EMBASE. The design followed PRISMA (Preferred Reporting Items for Systematic Reviews and Meta-Analyses) guidelines and was registered on PROSPERO (CRD42021271223).

### 2.2. Study Selection

**Figure 1:**
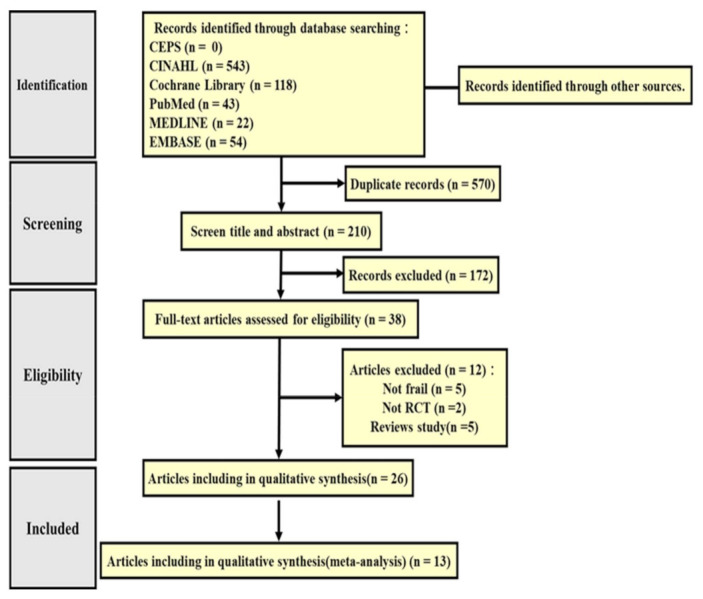
RISMA flow diagram.

In this study, our target population was the frail elderly living in the community. We used the PICO framework to search for the following terms: (1) Population: community elderly, community-dwelling older adults, frailty, frail; (2) Intervention: physical activity, exercise, fitness; (3) Comparison: usual care or no intervention; (4) Outcome: frailty status and different physical performance. The exclusion criteria were as follows: (1) the elderly participants were not living in the community; (2) the studies did not describe the efficacy of physical training on physical performance; (3) the study was a non-randomized controlled trial; (4) the article was not a full-text document published in 2017–2021. We excluded duplicate and ineligible studies. This research sample consisted of 13 studies (Figure 1).

### 2.3. Risk of Bias Appraisal

The research was performed according to the Cochrane Handbook for Systematic Reviews of Interventions, and the risk of bias was independently assessed by Li and Hsieh. The risk of bias focused on (1) random sequence generation (selection bias), (2) allocation concealment (selection bias), (3) blinding of participants and personnel (performance bias), (4) blinding of outcome assessment (detection bias), (5) incomplete outcome data (attrition bias), (6) selective reporting (reporting bias) and (7) other biases [17]. If the assessed item was clearly and fully described, the study was considered as having a “low risk”. Conversely, if it was not described or considered as having a “high risk”, it was described as unclear or incomplete and considered as having an “unclear risk”. The third author, Tallutondok, was responsible for resolving any disagreements about the risk of bias assessment.

### 2.4. Statistical Analysis

Meta-analysis was performed in RevMan 5.4.1. software (Cochrane Training site based in London, UK) [18]. A conservative random-effects meta-analysis model (*p* < 0.05) was used under the effects of heterogeneity between research designs in the study intervention dose, frequency, duration and result measurement. The 95% confidence interval (CI) was used for accuracy to identify and avoid underestimating the variability.

## 3. Results

### 3.1. Baseline Characteristics of the Selected Studies

The initial search retrieved a total of 780 studies. Of these, 210 studies were duplicates and thus removed. After assessing the remaining studies for suitability and completeness, 172 were excluded, and another 12 were excluded because they were not full texts or were ineligible due to incompatible criteria, which left 26 studies that satisfied the systematic review criteria. Finally, 13 studies were included in the meta-analysis (Table 1).

### 3.2. Risk of Bias Assessment

**Figure 2:**
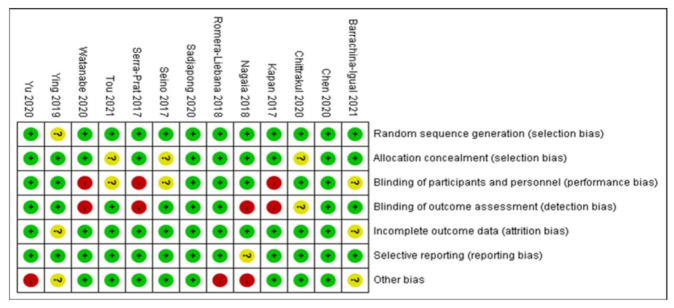
Risk of bias summary of all included studies.

Thirteen studies were assessed for risk of bias (Figure 2) [13,15,19,20,21,22,23,24,25,26,27,28,29]. All articles that provided detailed information of random sequence generation were considered low-risk [13,15,19,20,21,22,23,24,25,26,27,28,29]. Ten studies that used sealed envelopes or were double-blind trials that also had adequate allocation concealment were considered low-risk [13,15,19,21,22,23,24,25,26,27]. Seven studies with participants and personnel that were single blinded were considered low-risk [13,19,20,22,23,25,26]. Eight studies that clearly described blinding of the outcome assessments were labeled low-risk [13,19,23,25,26,27,28,29]. Two studies which did not specify whether they adopted intention-to-treat (ITT) analyses were labeled unclear-risk [25,27]. Only one article, which provided insufficient information on the main outcome, was considered high-risk [22]. Eight studies that clearly described sources of funding and avoidance of supervision were low-risk [15,19,20,21,23,24,28,29].

### 3.3. Study Characteristics

The included studies were from Austria [21], China [26], Japan [15,22,28], Singapore [29], Spain [13,24,27], Taiwan [19,25] and Thailand [20,23]. Together, the studies recruited a total of 3176 participants, who were randomized into experimental groups (*n* = 1585) and control groups (*n* = 1591). The number of participants in each group ranged from 20 to 549.

#### 3.3.1. Intervention Design

In the intervention designs, four studies had only physical training (PT) as the intervention [20,23,25,29]. The most common form in the other nine studies was PT combined with additional components [13,15,19,21,22,24,26,27,28]. Seven of the studies combined PT and nutrition components [13,15,19,21,22,24,28]. Three included PT and cognition components [13,19,26]. Seven combined PT with physical practice at home [15,19,22,23,24,28,29]. Two studies used technology for PT interventions [25,26].

#### 3.3.2. Types of Physical Training

Different types of PT were used in the studies. The largest group, 12 studies, focused on strength training [15,19,20,21,22,23,24,25,26,27,28,29], which included resistance work, bicycle training, squats, presses with elastic resistance bands, leg presses, knee extensions, leg abduction, and seated rowing. Eight focused on balance training [19,20,21,23,24,25,27,29]. This type of balance training design include dynamic balance training, static balance training, and balance impairment training. Seven studies included aerobic training [13,19,23,24,25,26,27] such as walking, stepping and cycling. Two studies involved flexibility training [19,27].

#### 3.3.3. Dose-Response of Physical Training: Dose

The doses of PT also varied. Nine studies had individual physical training sessions of about 60 min [13,20,23,24,25,26,27,28,29]. These studies were from China, Japan, Singapore, Spain, Taiwan, and Thailand. Two studies had PT session lengths of less than 60 min [19,21]. The studies were from Austria and Taiwan. Only one article (from Japan) had sessions of 90 min [15].

#### 3.3.4. Dose-Response of Physical Training: Frequency

The frequency of PT sessions also varied in the studies. Seven studies––from Austria, China, Japan, Singapore, and Spain––set PT sessions at twice per week [13,21,22,26,27,28,29] and three studies, from Taiwan and Thailand, had PT sessions 3 times per week [20,23,25]. One had a schedule of 4 times per week [24] and another, once per week [15]. In one study, the PT changed from coached to solo exercise [19]. These studies were from Japan, Spain, and Taiwan.

#### 3.3.5. Dose-Response of Physical Training: Duration

The physical training programs varied in length. In nine studies––from Austria, China, Japan, Singapore, Spain, Taiwan, and Thailand––they lasted 12 weeks [15,19,20,21,25,26,27,28,29]. Two studies, from Japan and Thailand, involved continuous PT for 24 weeks. [22,23]. One had a PT program of 6 weeks [13]; the other for 12 months [24]. These programs were from Spain.

#### 3.3.6. Adverse Events

Eight studies recorded adverse events [13,21,22,24,25,26,28,29]. Seven of them reported no health problems related to the intervention [13,22,24,25,26,28,29], while one article reported a single case of back pain as an adverse effect, which was from Austria [21].

### 3.4. Outcome Measures

#### 3.4.1. Effectiveness on Frailty

**Figure 3:**
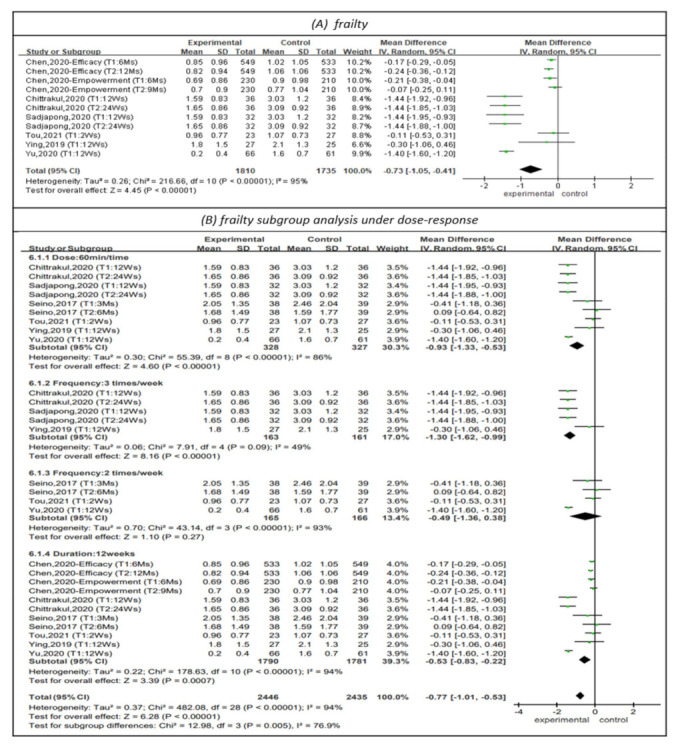
Forest plot of effects on frailty and subgroup analysis for dose-response. The size of the green square represents the weight of the study in the meta-analysis. The rhombus represents the combined OR. OR = odds ratio.

The Cardiovascular Health Study (CHS) criteria were used to represent frailty status in six studies, as shown in Figure 3 [19,20,23,25,26,29]. The CHS defines frailty status as the presence of any one of five frailty criteria (muscular weakness, slow walking speed, fatigability, sedentary lifestyle, and unintentional weight loss). The number of the criteria represented the severity of frailty. The results showed that physical training had a positive impact on frailty status (MD = −0.73, 95% CI (−1.05, −0.41), Z = 4.45, *p* < 0.01). In addition, subgroup analysis of studies with PT sessions lasting 60 min [20,23,25,26,28,29] indicated that such sessions had a positive impact on frailty status (MD = −0.93, 95% CI (−1.33, −0.53), Z = 4.60, *p* < 0.01). The subgroup analysis of studies with PT frequencies of 2 [26,28,29] and 3 times per week [20,23,25] indicated that physical training at the latter frequency had a positive impact on frailty status (MD = −1.30, 95% CI (−1.62, −0.99), Z = 8.16, *p* < 0.01). Subgroup analysis of PT programs lasting 12 weeks [19,20,25,26,28,29] indicated that physical training for this duration had a positive impact on frailty status (MD = −0.53, 95% CI (−0.83, −0.22), Z = 3.39, *p* < 0.01).

#### 3.4.2. Effectiveness on Physical Performance

**Figure 4:**
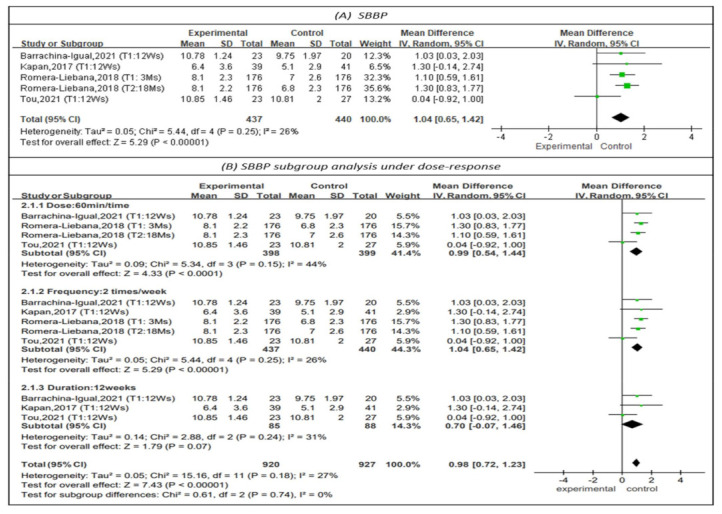
Forest plot of effects on SBBP and subgroup analysis for dose-response. The size of the green square represents the weight of the study in the meta-analysis. The rhombus represents the combined OR. OR = odds ratio.

The Short Physical Performance Battery (SPPB) was used to represent physical performance in four studies, as shown in Figure 4 [13,21,27,29]. The SPPB assesses balance, gait speed, and repeated chair stands. For each domain, a score of 0–4 was calculated, with higher scores indicating better functional performance. The results showed that physical training had a positive impact on physical performance (MD = 1.04, 95% CI (0.65, 1.42), Z = 5.29, *p* < 0.01). The I^2^ value was 26%. In addition, the subgroup analysis of studies with PT sessions of 60 min [13,27,29] indicated that sessions of this length had a positive impact on physical performance (MD = 0.99, 95% CI (0.54, 1.44), Z = 4.33, *p* < 0.01). The I^2^ value was 44%. The subgroup analysis of studies with PT sessions 2 times per week [13,21,27,29] showed that PT at this frequency had a positive impact on physical performance (MD = 1.04, 95% CI (0.65, 1.42), Z = 5.29, *p* < 0.01). The I^2^ value was 26%. Subgroup analysis of a PT program duration of 12 weeks [21,27,29] did not show that this duration of PT had a positive impact on physical performance (MD = 0.70, 95% CI (−0.07, 1.46), Z = 1.79, *p* = 0.07).

#### 3.4.3. Effectiveness on Upper Limb Strength

**Figure 5:**
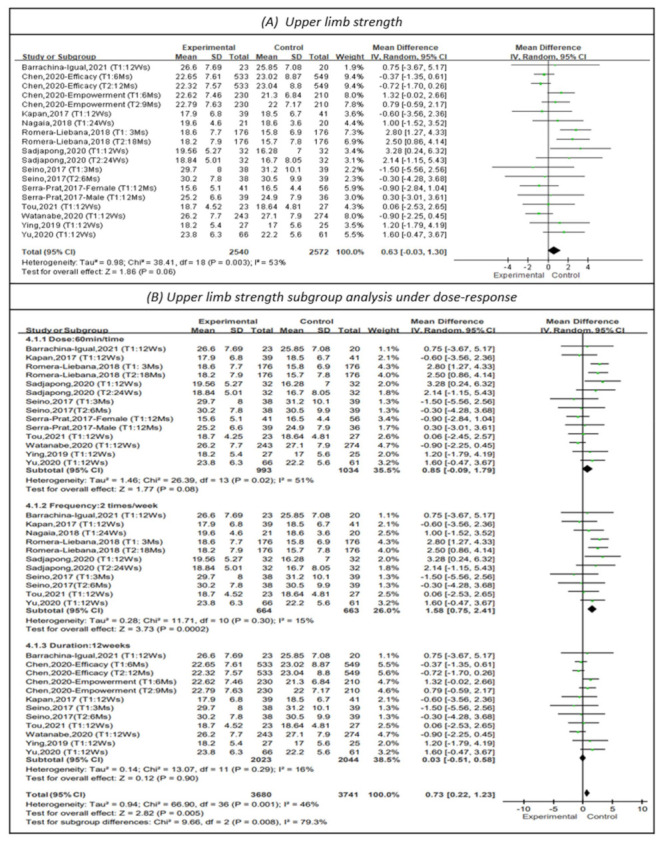
Forest plot of effects on upper limb strength and subgroup analysis for dose-response. The size of the green square represents the weight of the study in the meta-analysis. The rhombus represents the combined OR. OR = odds ratio.

Handgrip strength was measured by a dynamometer to represent upper limb strength in 12 studies, as shown in Figure 5 [13,15,19,21,22,23,24,25,26,27,28,29]. The results indicated a favorable but not statistically significant effect on upper limb strength (MD = 0.63, 95% CI (−0.03, 1.30), Z = 1.86, *p* = 0.06). In addition, subgroup analysis of studies with PT sessions of 60 min [13,15,21,23,24,25,26,27,28,29] did not show that 60-min sessions improved upper limb strength (MD = 0.85, 95% CI (−0.09, 1.79), Z = 1.77, *p* = 0.08). The subgroup analysis of studies with PT frequencies of 2 times per week [13,21,22,23,26,27,28,29] indicated that physical training at this frequency had a positive impact on upper limb strength (MD = 1.58, 95% CI (0.75, 2.41), Z = 3.37, *p* < 0.01). The I^2^ value was 15%. Subgroup analysis of studies that had continuous 12-week programs [15,19,21,25,26,27,28,29] did not indicate that such programs improved upper limb strength (MD = 0.03, 95% CI (−0.51, 0.58), Z = 0.12, *p* = 0.90).

#### 3.4.4. Effectiveness on Lower Limb Strength

**Figure 6:**
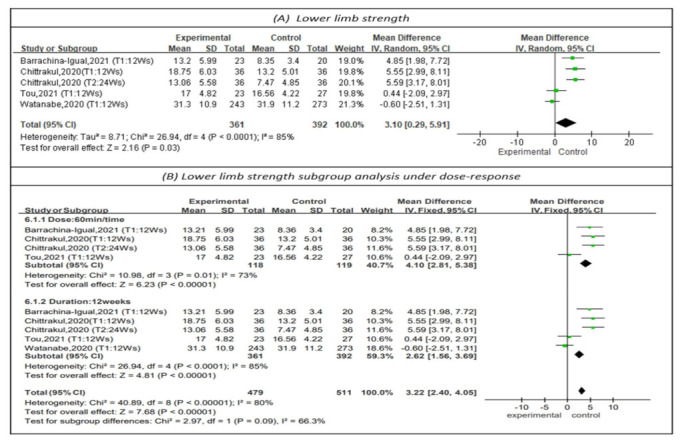
Forest plot of effects on lower-limb strength and subgroup analysis for dose-response. The size of the green square represents the weight of the study in the meta-analysis. The rhombus represents the combined OR. OR = odds ratio.

Knee extension was measured to represent lower limb strength in four studies, as shown in Figure 6 [15,20,27,29]. The results showed that physical training had a positive impact on lower limb strength (MD = 3.10, 95% CI (0.29, 5.91), Z = 2.16, *p* < 0.05). In addition, subgroup analysis of studies with sessions of 60 min [20,27,29] showed that sessions of this length had a positive impact on lower limb strength (MD = 4.10, 95% CI (2.81, 5.38), Z = 6.23, *p* < 0.01). Subgroup analysis could not be performed on the frequency of the dose-response due to insufficient data. The subgroup analysis of studies with continuous 12-week PT programs [20,27,29] showed that physical training for 12 weeks had a positive impact on lower limb strength (MD = 2.62, 95% CI (1.56, 3.69), Z = 4.81, *p* < 0.01).

#### 3.4.5. Effectiveness on Balance

**Figure 7:**
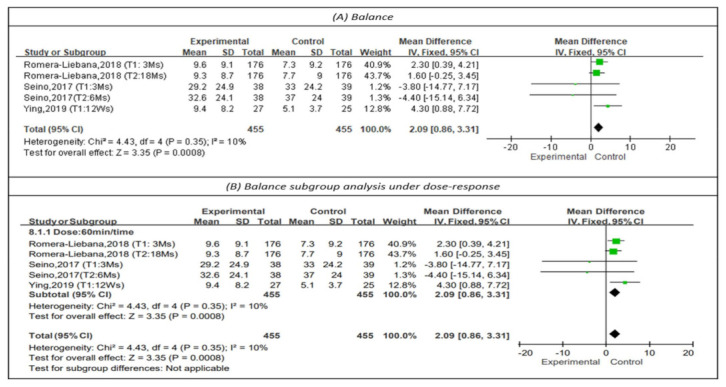
Forest plot of effects on balance and subgroup analysis for dose-response. The size of the green square represents the weight of the study in the meta-analysis. The rhombus represents the combined OR. OR = odds ratio.

Single leg stance was measured to represent balance in three studies, as shown in Figure 7 [13,25,28]. Longer single-leg standing times indicated better balance. The results showed that physical training had a positive impact on balance (MD = 2.09, 95% CI (0.86, 3.31), Z = 3.35, *p* < 0.01). The I^2^ value was 10%. In addition, subgroup analysis for sessions of 60 min [13,25,28] showed that such sessions had a positive impact on balance (MD = 2.09, 95% CI (0.86, 3.31), Z = 3.35, *p* < 0.01). The I^2^ value was 10%. No subgroup analysis could be performed on the frequency and duration of the dose-response due to insufficient literature.

#### 3.4.6. Effectiveness on Mobility

**Figure 8:**
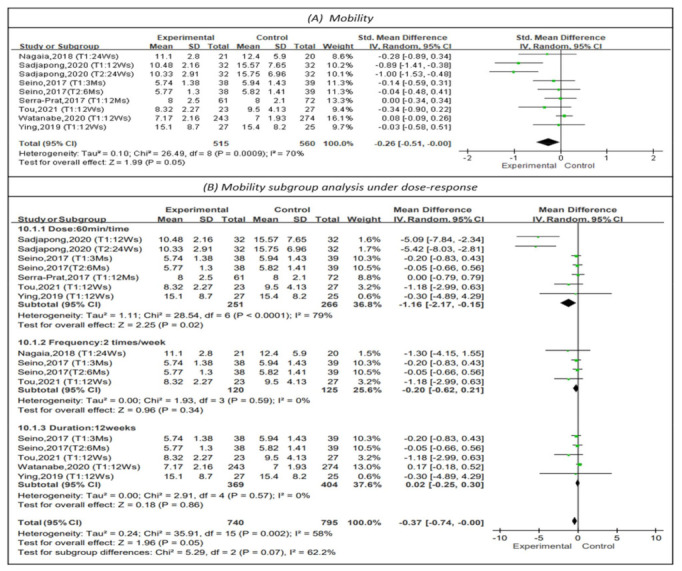
Forest plot of effects on mobility and subgroup analysis for dose-response. The size of the green square represents the weight of the study in the meta-analysis. The rhombus represents the combined OR. OR = odds ratio.

The timed up-and-go (TUG) was measured to represent mobility in seven studies, as shown in Figure 8 [15,22,23,24,25,28,29]. A shorter TUG time indicates better mobility. The results showed a favorable but not statistically significant effect on mobility (MD = −0.26, 95% CI (−0.51, 0.00), Z = 1.99, *p* = 0.05). In addition, subgroup analysis for sessions of 60 min [23,24,25,28,29] indicated that such sessions had a positive impact on mobility (MD = −1.16, 95% CI (−2.17, −0.15), Z = 2.25, *p* < 0.05). Subgroup analysis of studies with a PT frequency of 2 times per week [22,28,29] did not support that physical training at this frequency improved mobility (MD = −0.20, 95% CI (−0.62, 0.21), Z = 0.96, *p* = 0.34). Subgroup analysis of a PT program duration of 12 weeks [15,25,28,29] did not show that this duration of PT improved mobility (MD = 0.02, 95% CI (−0.25, 0.30), Z = 0.18, *p* = 0.86).

## 4. Discussion

### 4.1. The Efficacy of Physical Training in the Community-Dwelling Elderly

Clinical practice, academic research and policy development have begun to focus on the community-dwelling frail elderly due to the aging of the global population. Because frailty in the elderly will affect physical performance [3], we integrated recent evidence and found that physical training had a positive impact on frailty status, physical performance, lower limb strength and balance. Kidd et al. performed a systematic review and found that physical training was effective in improving physical performance in the community-dwelling frail elderly [9]. The systematic review and meta-analysis of Zhang et al. indicated that balance and mobility were significantly improved by physical training [6]. Jadczak et al. performed an umbrella review and also found that physical training was effective in improving physical performance and muscle strength [30]. The findings of our review are similar to those in the previous literature, but the previous literature did not include frailty status. In clinical application and policy formulation, we recommend including physical training for the frail elderly in the community, which can improve their frailty status and physical performance. Although physical training interventions have several positive effects on the frail elderly, there are some potential risks of non-significant effects under imperfect dose-response during implementation. If physical training is excessive or insufficient, it will not improve the health of the community-dwelling frail elderly. Therefore, we performed subgroup analyses on dose, frequency, and duration to analyze the dose-response of physical training. The evidence-based dose-response aims to benefit from physical training effects while circumventing potential risks of non-significant effects.

### 4.2. Dose-Response Efficacy of Physical Training on Frailty Status

In recent years, the impact of dose-response in physical training on the frail elderly has not been evaluated [7]. In our systematic review, pooled analysis indicated that physical training sessions of 60 min, 3 times per week for 12 weeks, could mitigate frailty. To date, no empirical studies have investigated which optimal dose-response format of physical training has the best frailty therapy effect in clinical practice. Our findings demonstrate preliminary evidence that an optimal dose-response in physical training shows potential as frailty improves intervention. Therefore, the dose-response should be recommended as a requirement for a physical training program for the frail elderly.

### 4.3. Dose-Response Efficacy of Physical Training on Physical Performance

#### 4.3.1. Dose-Response Efficacy on Physical Performance

Gaps in the dose-response effect of physical training designs may be the result of evidence, habitation or social culture, which impact the effectiveness of the intervention. Currently, the results of a meta-analysis have focused on the physical performance of the frail elderly. Jadczak et al. found that physical training sessions of 20–90 min, 1–5 times per week continuously for 2.5–18 months improved physical performance in this population [30]. We found that physical training sessions of 60 min, 2 times per week improved physical performance. Our findings identified evidence for the beneficial effect of an optimal dose-response in physical training on the physical performance of the frail elderly. Simultaneously, we narrowed the dose-response range in physical training.

#### 4.3.2. Dose-Response Efficacy on Muscular Strength

In recent years, studies have confirmed the beneficial muscular strength effects of dose-response in physical training on the frail elderly. Zhang et al. found that physical training programs of less than 12 weeks could improve upper-limb strength [6], and Jadczak et al. found that physical training sessions of 20–90 min, 2–5 times per week continuously for 2.5–9 months improved muscular strength [30]. Lopez et al. found that physical training 1–6 times per week improved muscle strength and muscle power [11]. However, we found that physical training 2 times per week improved upper-limb strength, while sessions of 60 min for 12 weeks improved lower-limb strength. Our results were different from other recent systematic reviews because we identified evidence of a beneficial effect of optimal dose-response in physical training on the muscular strength of the frail elderly.

#### 4.3.3. Dose-Response Efficacy on Balance

There are several explanations for the positive effects of dose-response range in physical training on the balance of the frail elderly. Zhang et al. found that physical training programs lasting less than 12 weeks improved balance [6], and Jadczak et al. found that physical training sessions of 20–75 min, 3 times per week continuously for 2.5–18 months improved balance [30]. We found that physical training sessions of 60 min improved balance. Although the dose-response in physical training can exert a positive impact on the balance of the frail elderly, the uncleared dose-response range will make the physical activity program difficult. In addition, due to insufficient evidence of dose-response in physical training in our included studies, further research should be conducted to explore the accuracy of frequency and duration parameters but not dose parameters.

#### 4.3.4. Dose-Response Efficacy on Mobility

Regarding the effect of dose-response on mobility, some researchers believe that it leads to increased mobility in physical training. Jadczak et al. found that sessions of 26–90 min, 1–7 times per week for 5 weeks to 18 months improved mobility [30]. We found that physical training sessions of 60 min could improve mobility. Similarly, the dose-response in physical training exerted a positive impact on the mobility of the frail elderly. To avoid controversy, the dose-response range should be clearly defined. Therefore, research is also needed to increase the understanding of the optimal frequency and duration parameters for mobility to further design a complete frailty care program.

### 4.4. Strengths and Limitations

The advantages of our systematic review are as follows. First, it was based on PICO procedures. Second, it retrieved objective results and clarified causality, which is a limitation of RCTs. Third, all the studies were assessed for risk of bias according to the Cochrane Handbook for Systematic Reviews of Interventions, in which the authors used a strict quality assessment of the studies and a systematic combination of findings after serious evaluations. Fourth, participants with different frailty statuses were included in the analysis. This study is the first systematic review and meta-analysis of RCTs that explored the effectiveness of frailty status in the elderly. Simultaneously, we focused on the dose, frequency, and duration parameters of physical training. However, several limitations of our systematic review may explain the differences in the research outcomes. First, the included studies were all published in English. Second, no non-randomized controlled trials or grey studies were included; in addition, the different evaluation tools and the limitation to a period of five years may have affected the results. Third, some unblinded studies were considered high-risk and lacked adequate allocation concealment to confirm whether the randomized control affected the outcome of physical training, thereby limiting the conclusions. Fourth, the different types of physical training included multiple methods and components, which may have affected the outcome and interfered with the dose-response efficacy of the physical training. Finally, in some studies, the statistical power was low because of a small sample size. We supposed that the above limitations may explain the differences between this review and others, but the current review still provides new evidence on the optimal dose-response effect of physical training in the community-dwelling frail elderly.

## 5. Conclusions

Our systematic review integrated recent RCTs to determine the optimal dose–response relationship of physical training in community-dwelling frail elderly. If the optimal dose-response of physical training is added to the health promotion program, it can mitigate frailty and increase physical function, and it can also reduce the risk of complications or injury. Future research should consider the effective and clear dose-response effects of physical training and conduct long-term follow-up. Community care stations or day care centers should focus on the dose-effect of physical training and add such information to the health promotion program. Practice based on theory should guide interventions for the elderly to improve the health of community-dwelling frail elderly.

## Figures and Tables

**Table 1:** Characteristics of the included studies.

Study	Sample (I/C)	Experimental Group	Control Group	Follow Time	Outcome Measures
Barrachina-Igual, 2021	43 (23/20)	Resistance training combined with self-massage for myofascial release. Dose-response: about 60 min/time (dose), 2 times/week (frequency), total 12 weeks (duration).	Unclear	T1: 12 Wk	Physical frailty Muscle function Functional performance
Chen, 2020	Efficacy: 1082 (549/533) Empowerment: 440 (230/210)	Efficacy Study: physical exercise, cognitive training and nutritional counseling. Dose-response: 45 min/time (dose). The sessions were curtailed and home practiced alone (frequency), total 12 weeks (duration). Empowerment Study: Efficacy version redesigned and additionally empowered. Dose-response: 45 min/time (dose), the sessions were completed at home and curtailed and monitored for amount of practice (frequency), total 12 weeks (duration).	The Efficacy Study compared telephone consultation. The Empowerment Study compared the standard Efficacy Study.	Efficacy T1: 6 Mo T2: 12 Mo Empowerment T1: 6 Mo T2: 9 Mo	CHS frailty score, Gait speed Grip strength
Chittrakul, 2020	72 (36/36)	Proprioception training, muscle strength training, reaction time exercise training with auditory cues, and postural balance training. Dose-response: 60 min/time (dose), 3 times/week (frequency), total 12 weeks (duration).	The control group received flexibility exercise training three times each week of the program.	T1: 12 Wk T2: 24 Wk	Knee extension strength
Kapan, 2017	80 (39/41)	Strength exercise programs supplemented with a nutrition. Dose-response: 35 min/time (dose), 2 times/week (frequency), total 12 weeks (duration).	The control group engaged in cognitive practice.	T1: 12 Wk	Physical functioning Handgrip strength Physical activity level
Nagaia, 2018	41 (21/20)	Resistance training. Dose-response: 2 times/week (frequency), total 24 weeks (duration).	The control group received resistance training.	T1: 24 Wk	Frailty status Physical function Muscle strength
Romera-Liebana, 2018	352 (176/176)	Physical activity, high-protein nutritional shake, memory workshops and medication review. Dose-response: 60 min/time (dose), 2 times/week (frequency), total 6 weeks (duration).	The control group received usual care.	T1: 3 Mo T2: 18 Mo	SPPB Strength by handgrip Functional reach Balance
Sadjapong, 2020	64 (32/32)	Aerobic training, resistance training, balance training and home practice. Dose-response: 60 min/time (dose), 3 times/week (frequency), total 24 weeks (duration).	The control group received usual care.	T1: 12 Wk T2: 24 Wk	Physical performance Hand strength Balance Endurance Frailty scores
Seino, 2017	77 (38/39)	Resistance exercise, nutritional or psychosocial programs and home practice. Dose-response: 60 min/time (dose), 2 times/week (frequency), total 12 weeks (duration).	The control group continued with their daily activities.	T1: 3 Mo T2: 6 Mo	Frailty status Physical function
Serra-Prat, 2017	172 (80/92)	Nutritional assessment and physical activity program (strength, balance and coordination exercises). Dose-response: about 60 min/time (dose), 4 times/week (frequency), total 12 months (duration).	The control group received usual care.	T1: 12 Mo	Frailty status Hand grip
Tou, 2021	57 (27/30)	Progressive power, balance exercises and home practice). Dose-response: 60 min/time (dose), 2 times/week (frequency), total 12 weeks (duration).	The control group continued with the exercise program.	T1: 12 Wk	Physical Function Frailty Status
Watanabe, 2020	517 (243/274)	Low-load resistance exercises, 2500 steps/day, oral functional care, a nutritional guide and home monitored practice. Dose-response: 90 min/time (dose), 1 time/week (frequency), total 12 weeks (duration).	The control group carried out the program by themself at home.	T1: 12 Wk	Physical functions: grip strength, knee extension strength, walking speeds, TUG, five-time chair standing test, 30 s chair stands, functional reach test, chair stepping test and a vertical jump index Physical activity
Ying-Yi, 2019	52 (25/27)	Tai-Chi, resistance and aerobic combination training and balance training. Dose-response: 60 min/time (dose), 3 times/week (frequency), total 12 weeks (duration).	The control group received a combined exercise program.	T1: 12 Wk	Frailty status SPPB Back scratch Chair sit and reach 30-s sit-to-stand 2-min step Single leg stance Functional reach Timed up and go Walking speed Grip strength
Yu, Tong, 2020	127 (66/61)	Multicomponent Frailty Prevention Program combined with exercise, computer-assisted cognitive training, and board game activities. Dose-response: 60 min/time (dose), 2 times/week (frequency), total 12 weeks (duration).	The control group received usual care.	T1: 12 Wk	Frailty Hand-grip strength Muscle endurance Balance Gait speed

## Data Availability

All the data underlying the findings are fully available without restriction. All relevant data is within this study.
